# Sample-Based Vegetation Distribution Information Synthesis

**DOI:** 10.1371/journal.pone.0134009

**Published:** 2015-08-07

**Authors:** Chanchan Xu, Gang Yang, Meng Yang

**Affiliations:** 1 School of Information Science and Technology, Beijing Forestry University, Beijing, China; 2 School of Animation and Digital Arts, Communication University of China, Beijing, China; Chinese Academy of Forestry, CHINA

## Abstract

In constructing and visualizing a virtual three-dimensional forest scene, we must first obtain the vegetation distribution, namely, the location of each plant in the forest. Because the forest contains a large number of plants, the distribution of each plant is difficult to obtain from actual measurement methods. Random approaches are used as common solutions to simulate a forest distribution but fail to reflect the specific biological arrangements among types of plants. Observations show that plants in the forest tend to generate particular distribution patterns due to growth competition and specific habitats. This pattern, which represents a local feature in the distribution and occurs repeatedly in the forest, is in line with the “locality” and “static” characteristics in the “texture data”, making it possible to use a sample-based texture synthesis strategy to build the distribution. We propose a vegetation distribution data generation method that uses sample-based vector pattern synthesis. A sample forest stand is obtained first and recorded as a two-dimensional vector-element distribution pattern. Next, the large-scale vegetation distribution pattern is synthesized automatically using the proposed vector pattern synthesis algorithm. The synthesized distribution pattern resembles the sample pattern in the distribution features. The vector pattern synthesis algorithm proposed in this paper adopts a neighborhood comparison technique based on histogram matching, which makes it efficient and easy to implement. Experiments show that the distribution pattern synthesized with this method can sufficiently preserve the features of the sample distribution pattern, making our method meaningful for constructing realistic forest scenes.

## Introduction

The construction and visualization of large-scale forest scenes has become a hot research topic in computer graphics. In constructing a three-dimensional forest scene, the positions of all plants in the forest must first be generated. The distributions of plants in a real forest can be obtained via actual forest measurements. However, the vast areas and huge number of plants in a forest makes it difficult to obtain information on the entire forest distribution in this way. In current studies and applications, researchers generally use random methods to simulate the distribution of plants. These methods are easy to implement but fail to consider the specific biological distribution features and patterns of the forest.

It is believed that the distribution of plants in the forest is not completely random. Due to competition for resources and the specific growth characteristics of plant species, the distribution of plants tend to exhibit certain distribution features. For example, tall arbors prefer to maintain a distance, whereas shrubs prefer aggregated growth.

These distribution features often result in a vegetation distribution that presents a certain pattern, and the pattern occurs repeatedly in a certain scope of the forest [1]; thus, the complete forest scene exhibits certain repeatability and locality characteristics. These features are in line with the “locality” and “static” characteristics in the “texture data” [2]. Inspired by this observation, we propose a sample-based texture synthesis strategy for reconstruction of the distribution information for an entire forest. This so-called “sample-based texture synthesis” is a technique that synthesizes large patterns from a given small sample pattern using a selected algorithm. The sample pattern and the synthesized pattern appear to have the same distribution rules and similar appearance. In our work, the distribution of a large number of plants is mapped to the distribution of the same number of two-dimensional vector elements, namely, a two-dimensional vector pattern. In this manner, the reconstruction of forest distribution can be translated into a vector pattern synthesis problem.

**Fig 1:**
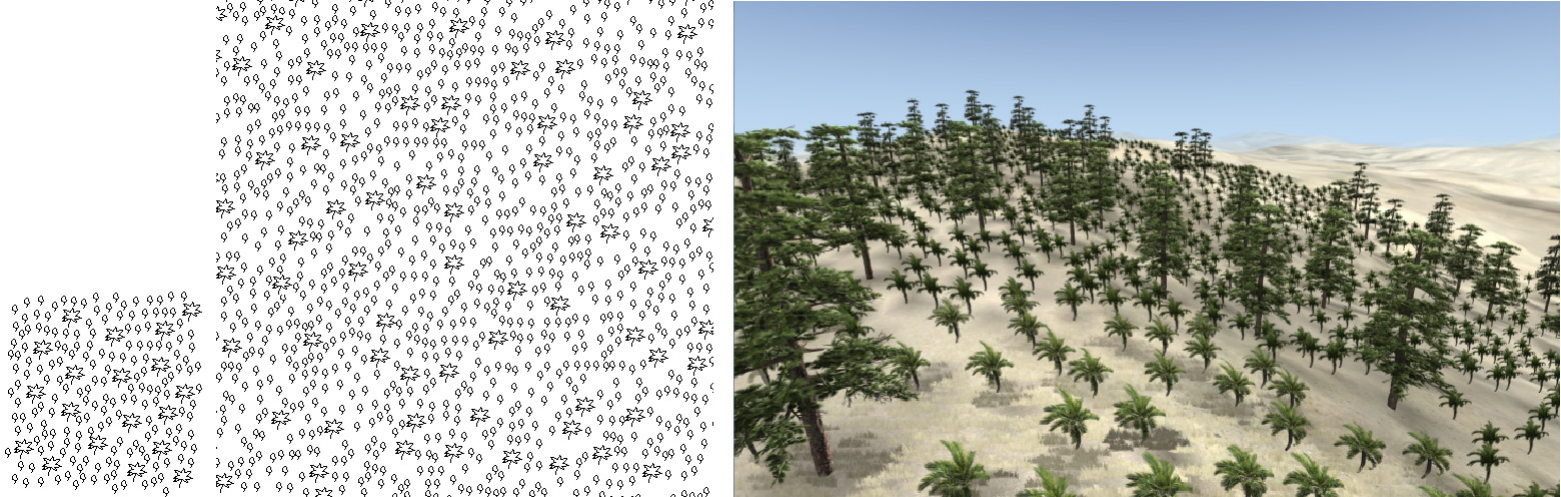
Plants distribution information synthesis and forest construction. Left: Sample pattern; Middle: Synthesized pattern based on the sample; Right: Forest scene constructed according to the synthesized distribution information.

The fundamental concepts of this approach are illustrated in Fig 1. We first obtain the distribution pattern of a small area of plants that represents the major distribution features of the forest. We refer to this small area of distribution as the “sample pattern” (Fig 1 left). Next, based on the sample pattern, we synthesize a large area of distribution information using our vector pattern synthesis method (Fig 1 middle). Finally, by loading the plant models, we can construct the 3D forest scene according to the distribution information (Fig 1 right).

In synthesizing the vector patterns, the key issue is how to describe the positional relationships between vector elements. To address this problem, we propose a histogram-based method that expresses the topology distribution of the vector elements. The neighborhood of an element is defined as a circle centered at the element that is divided into small grids. The neighborhood information is represented using a neighborhood histogram by calculating the numbers of neighborhood elements that lie in each grid. A search strategy based on the neighborhood and a new neighborhood comparison technique based on histogram matching are used in the synthesis phase.

This sample-based synthesis manner is highly suited to forestry data. The forest inventory and measurements are usually processed in one or several small blocks of forest area that represent the characteristics of the entire forest. Therefore, for a large area of forest, the vegetation information that we obtain represents only one or several small blocks of area. Our sample-based synthesis method can effectively use this information to generate virtual forest scenes that conform to the actual forestry data.

In summary, our main contributions are described as follows: 1) we propose a sample-based method for plant distribution information synthesis. Compared with previous methods, this method better reflects the forest distribution features and can be used to generate 3D virtual forest scenes that conform to the real forest scenes; 2) we propose a new vector pattern synthesis method based on “neighborhood histogram matching”, which has a higher efficiency than previous vector pattern synthesis methods and is easy to implement.

## Related Work

### A. Generation of the Forest Distribution

The common methods used to construct a large-scale forest scene primarily rely on a random distribution algorithm and interactive means to generate the position arrangement [3][4]. However, these methods cannot accurately reflect the distribution features of the plant communities. The random model that is often used for plant distribution is the Poisson disk distribution model. The Poisson disk distribution can ensure that a certain distance is maintained among plants but fails to reflect the more complicated features and patterns in the distribution. Certain researchers have attempted to use the Wang tiles approach in rapidly growing large-area forest synthesis [5][6] but could not take into account the actual distribution of plants because this approach also uses random models, i.e., the Poisson disk distribution, to generate the sample tiles. In this paper, we discard this random method. Instead, by mapping the distribution of plants as a two-dimensional vector element distribution pattern, we put forward a sample-based synthesis method for generating the large-scale vegetation distribution information of a forest.

### B. Vector Pattern Synthesis Based on Samples

Sample-based texture synthesis has been a hot topic in computer graphics for decades; its main task is to analyze the features of the given sample texture and automatically generate a larger texture that has visual similarity to the sample texture. The sample texture should possess locality and stationarity [2]. Locality means that a point value depends on its neighborhood, whereas stationarity means that this dependency is not related to the position of the point in the sample. Texture data that satisfy those two features can be described in a Markov Random Field and generated by a neighborhood matching method.

In the beginning, researchers focused on the synthesis method of images [7–13], but vector pattern synthesis has become a topic of interest in the last few years. Vector patterns are those patterns that consist of a number of vector elements whose type, location, and direction information are known. The synthesis methods must analyze and rebuild the arrangement rules among the vector elements. Barla et al. [14] first used a Lloyd method to obtain the initial distribution of a seed map and replace it with the space relationships between elements in the sample pattern. Hurtut et al. [15] employed a density function to define whether the random newly added element is valid for the synthesis pattern. These two methods attempt to capture the global features and present an overall similarity of the sample pattern but are only suitable for patterns that are mostly evenly distributed.

To capture the local distribution features, Barla et al. [16] use Delaunay triangulation to construct the topology distribution of the elements, and in this manner, the local neighborhood feature was obtained for pattern synthesis. Following this idea, Ijiri et al. [17] proposed a method based on local neighborhood comparison and local growth. Compared with methods based on global statistics, the local methods can sufficiently preserve the distribution features of the sample, and thus, we adopt this approach in our paper.

**Fig 2:**
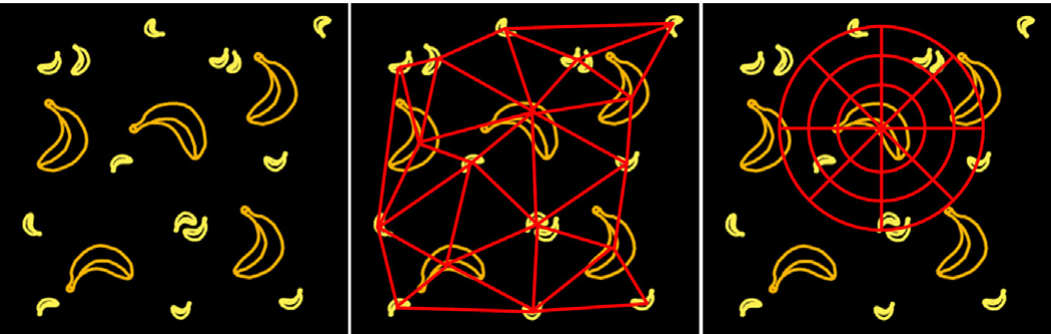
Explicit and implicit connection relationships between elements. Left: Sample pattern [10]; Middle: Delaunay triangulation; Right: Our method.

The definition and the comparison of elements’ neighborhood feature are key issues in synthesis methods based on neighborhood matching. The previous methods must construct the explicit connections between elements to indicate their neighborhood relationships. For example, the methods in [16][17][18] all use Delaunay triangulation to construct the spatial structure of the elements explicitly (shown in Fig 2b) and also must sort the neighboring elements for neighborhood matching. The triangulation and sorting process make the synthesis process some unstable and much more complicated. Liu et al. [18] attempted to describe the neighborhood relationships with a neighborhood coding method; however, this approach also must explicitly construct the connections and sort the elements.

We believe that no absolute relationships exist between elements, and thus there is no need to construct explicit connections. Inspired by this idea, we propose an implicit description method (Fig 2 right) based on the histogram description. Based on this foundation, the neighborhood searching and matching processes can be carried out by comparing the histograms. Compared with previous methods, our method can greatly simplify the neighborhood construction process and the neighborhood matching calculation. For these reasons, our method is superior.

In our paper, each plant is represented as an element, and the distribution of the plants can be expressed as a vector pattern. We generate the large-scale plant distribution by the synthesis of vector pattern.

## Overview

Our work includes three main stages: sample obtaining, analysis and pattern synthesis.

The sample data can be obtained through the field measurement or being generated by an interactive pattern design system.

During the sample analysis process, we first estimate the radius of the element’s neighborhood region. Next, we segment each element’s neighborhood area and calculate the histogram for the neighborhood.

During the synthesis process, in the first step, we select an element at random, place it at the center of the synthetic area, and copy its neighborhood elements to the corresponding positions in the synthesis area. The first element is known as the center element, and the center element and its neighborhood elements form an initial pattern in our synthesis pattern. Next, we extend the synthesis pattern in a step-by-step manner. In each extension, we choose the element nearest to the center element as the extending element, search for the matching element whose histogram is the most similar to that of extending element in the sample pattern, and copy the neighboring elements of the matching element to the synthesis area. By repeating the second step, we obtain our final results. We first demonstrate our method with one type of element and subsequently illustrate the synthesis of patterns with multiple types of elements.

## Sample Obtaining

By conducting a survey of a patch of realistic forest in the field, the information of trees’ position and species is obtained as the sample data. Then the position and species of each tree will be recorded and represented by a two-dimensional pattern, which will be used as the sample pattern for synthesis. Besides position and species, we can also get the DBH (Diameter at Breast Height), height and crown width for each tree in the field measurement. During the synthesis process, these data can be assigned to the trees in the synthesis pattern and used for setting the tree model’s size in constructing 3D forest scenes.

Normally it is not easy to obtain the realistic forest data. Therefore a pattern design system is developed to generate various sample patterns interactively. This system enables Users to drag elements from an element set to a certain area so as to design the sample pattern. The information for the elements, including position and types (id), can be obtained by the system during the design phase. Each element in the pattern can also be redesigned by the user using a brush tool.

## Sample Analysis

The neighborhood region of an element is defined as a circular space centered at this element. The space must be divided and calculated to obtain the distribution information for the sample pattern.

### A. Neighborhood Segmentation

**Fig 3:**
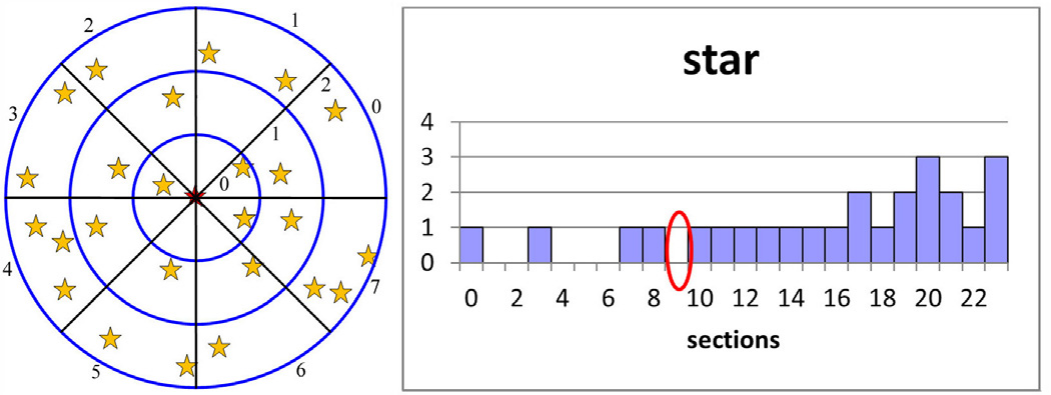
Neighborhood space partition (left) and the histogram generation (right).

We use a bundle of radial lines and a cluster of concentric circles to divide the neighborhood space into small grids. Currently, we use 8 radial lines and 3 concentric circles to divide the space into 24 grids (shown in Fig 3 left). A larger radius for the neighborhood might lead to better synthesis results but will result in heavier computation. In this paper, we define the radius of the smallest concentric circle as the shortest distance between elements in the sample pattern.

Each grid is represented by a set of parameters, i.e., (shell, sector). ‘Shell’ indicates the concentric circle level, and ‘sector’ indicates the sector index of the grid. As shown in Fig 3(left), the shell values are 0–2 from the inside out, and the sector values are 0–7 in a counterclockwise direction. If the position of a grid is identified as (1, 1), it means shell = 1, sector = 1.

### B. Histogram Generation

After neighborhood segmentation, we count the number of neighborhood elements lying in each grid. Each grid corresponds to one bin in the neighborhood histogram. The neighborhood histogram is generated by calculating the numbers for all the grids (shown in Fig 3 right). For example, no elements lie in the grid with shell = 1, sector = 1, and thus, the number of the corresponding histogram bin with index “shell*8+sector = 9” (circled in red) is 0.

## Pattern Synthesis

**Fig 4:**
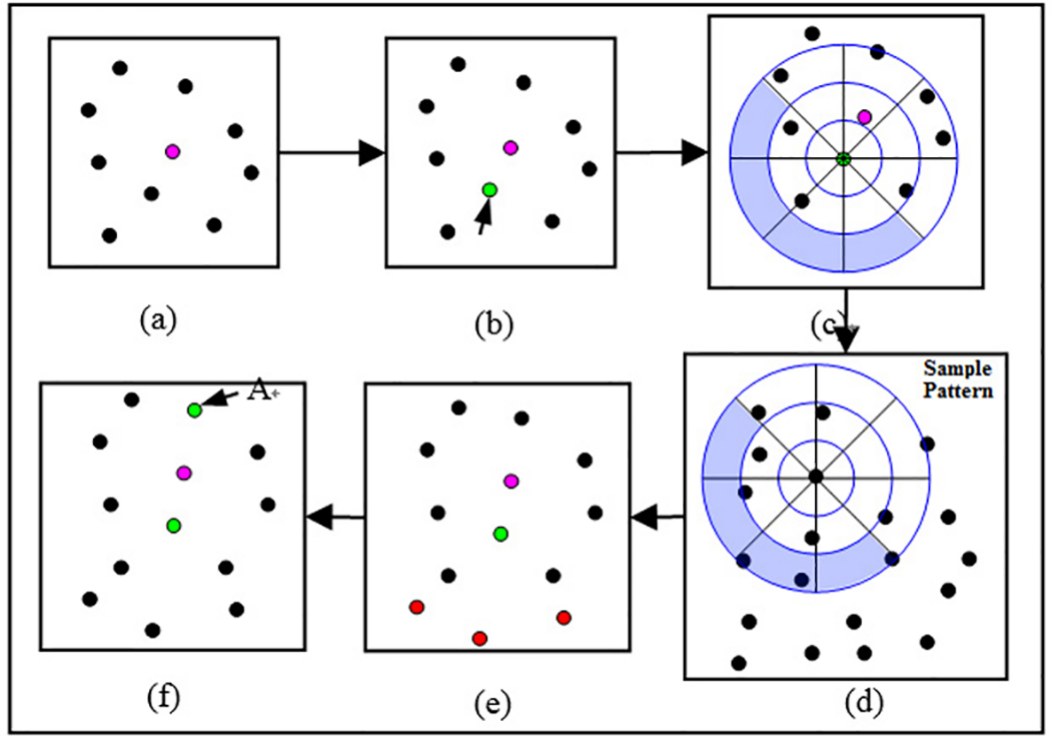
Synthesis process for a single type of element. (a) The synthesis pattern, with the pink element as the center element; b) the element closest to the center element is selected as the extending element (green element); (c) illustration of the determined grids (filled with blanks) and undetermined grids (filled with blue; (d) finding the matching element in the sample pattern according to the determined grids; (e) copying the corresponding neighborhood elements (the red elements) to the synthetic area; and (f) picking the next element (element A) for extension. The process is repeated to obtain the final synthesis results.

In the process of synthesis, we first choose an element from the sample pattern at random and place it at the synthesis area center, labeling it as the center element. The neighborhood elements of the center element are also copied to the corresponding positions in the synthesis area to form the initialized synthesis pattern. The final results are gradually extended from the initial pattern. In each extension, the element nearest to the center element in the synthesis pattern is set as the extending element. A histogram for the extending element must be calculated using the method introduced in the last Section B. According to this histogram, we search the sample pattern and find the matching element whose histogram is the most similar to that of the extending element. We copy the neighborhood elements of the matching element to the synthesis area to complete one extension. The major steps are shown in Fig 4.

To ensure that the extending element has sufficient neighborhood information for searching its matching element, we always choose the element closest to the center element as the extending element. Specifically, the selection standards are: 1) the distance to the center-element is the minimum in current synthesis pattern, and 2) the element has not been extended previously.

### A. Determination of Available Grids for the Extending Element

In the neighborhood space of the extending element, certain neighborhood grids are known, and the number of neighborhood elements can be determined, whereas others are undetermined because the extending element’s neighborhood is incomplete. The determined grids are used as a proof during the matching element search process. The undetermined grids will be replenished after the extension.

**Fig 5:**
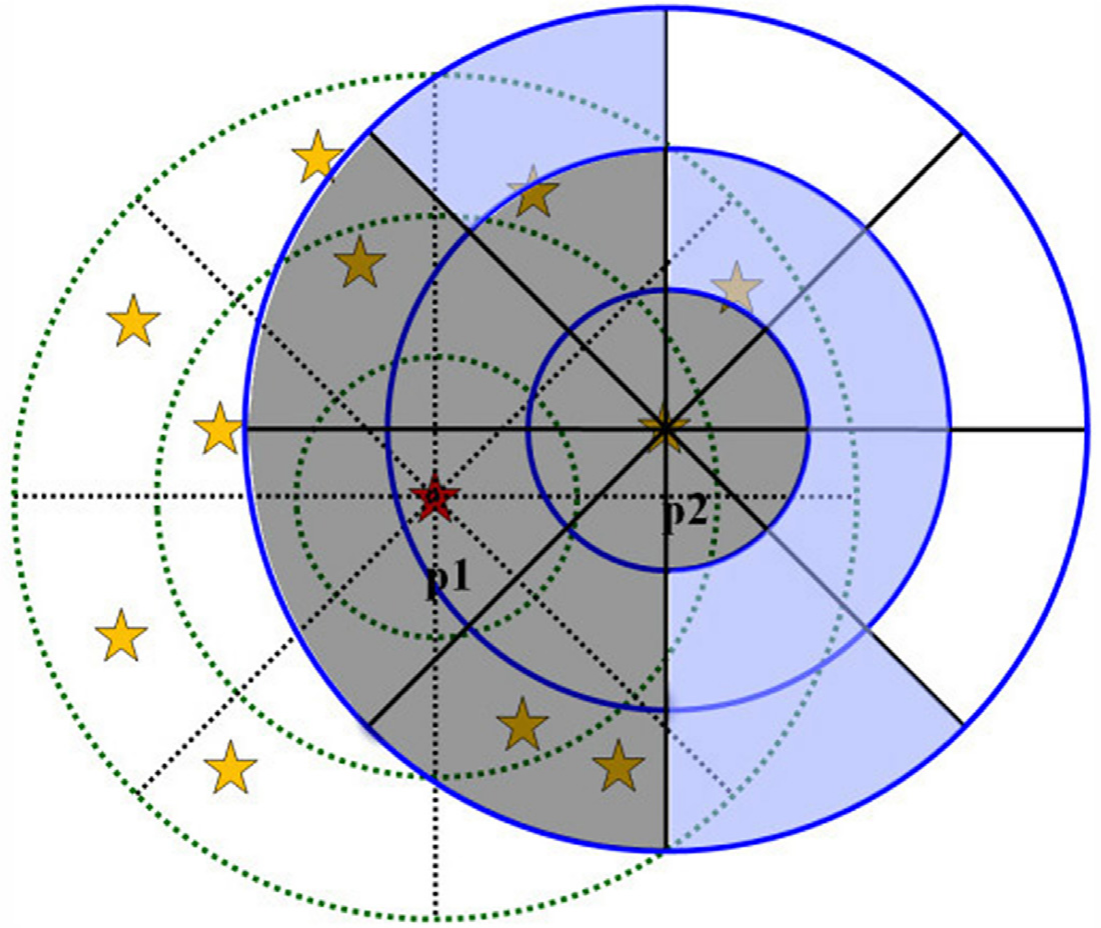
Decision on determined grids and undetermined grids. p1 is a former extended element and p2 is the current extending element.

To find these determined grids, we use a strategy that calculates the intersection area of the current extending element’s neighborhood space and the former extended element’s space. The grids lying in the overlap area are the determined grids, and the others are the undetermined grids. Fig 5 provides an illustration of the intersection of two neighborhood spaces.

It can be observed from Fig 5 that the grids lying in the overlap can be classified into two types: completely inside (filled with gray) and partially inside (filled with blue, known as undetermined grids). We make further decisions on these undetermined grids (filled with blue) using the representative point. A representative point is used to represent a grid. If the representative point lies in the former extended element’s neighborhood space, the corresponding grid is treated as a determined grid; otherwise, it is an undetermined grid.

**Fig 6:**
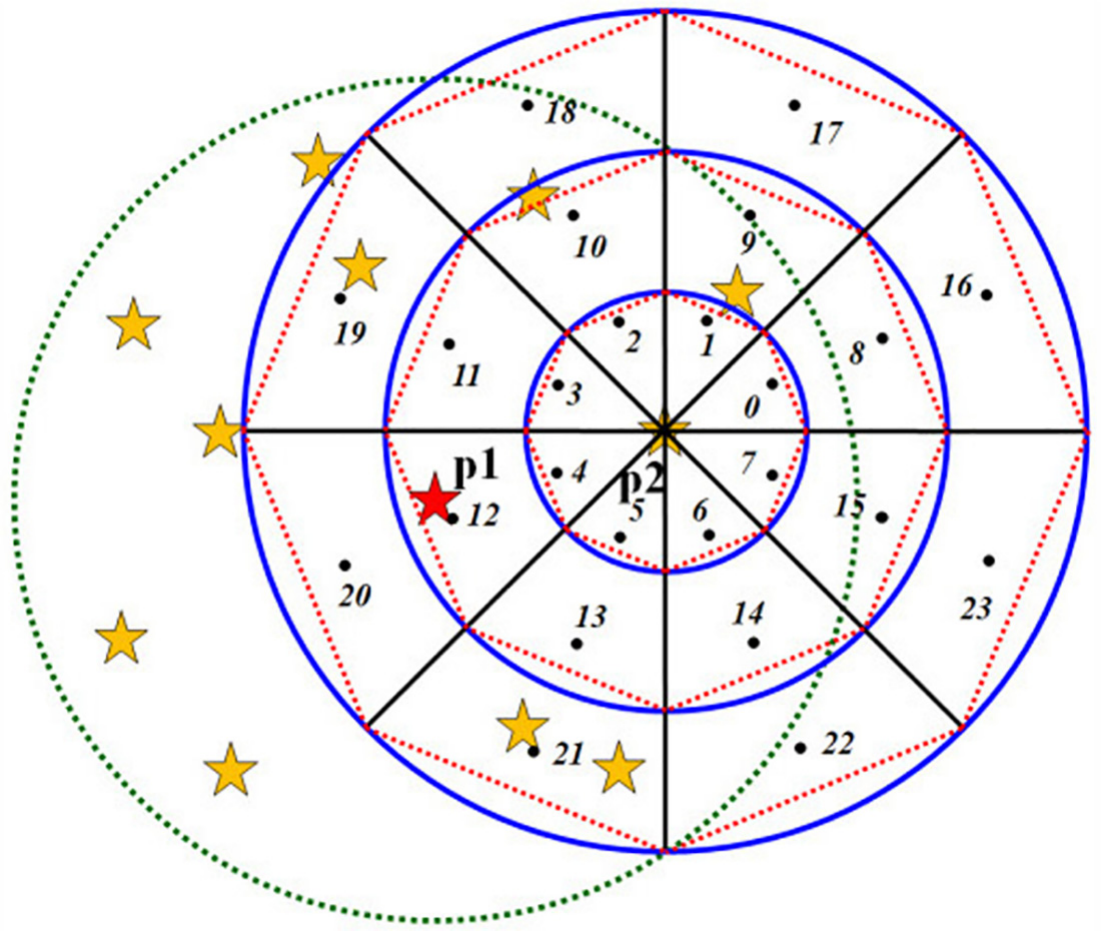
Determination of undetermined grids. p1 is a former extended element, and p2 is the current extending element.

As shown in Fig 6, point 18 is the representative point of grid (2, 2). Point 18 lies in the neighborhood space of p1, and thus, grid (2, 2) is a determined grid. The representative point 15 lies outside the neighborhood space of p1, and this grid (1, 7) is an undetermined grid.

If the extending-element intersects with more than one extended element, we consider the union of all intersections in deciding on the determined grids.

### B. Histogram Comparison

To quantify the distance of two histograms, a common solution uses the classic Euclidean distance, as shown in Formula 1. However, the Euclidean distance only considers the distance for grids with the same position and ignores the effects of the adjacent grids. We suggest that the adjacent grids also have relationships to the histogram distance. The closer the two grids are located to each other, the stronger their correlation will be. Therefore, a similarity matrix A is added to the classic Euclidean distance function to reflect the correlation relationships among grids, as shown in Formula 2. The component aij of matrix A represents the correlation relationship of the components i and j in the underlying vector space.

$$d_{euclid}^{2}\left(p,q\right)=\left(p-q\right)\cdot {\left(p-q\right)}^{T}={\sum_{i=1}^{N}\left(p_{i}-q_{i}\right)}^{2}$$ (1)

$$d_{A}^{2}\left(p,q\right)=\left(p-q\right)\cdot A\cdot {\left(p-q\right)}^{T}={\sum_{i=1}^{N}\sum_{j=1}^{N}\left(p_{i}-q_{i}\right)}^{2}$$ (2)

Where *a*_*ij*_ = *e* ^−*σd*(*i*,*j*)^,*d*(*i*,*j*) = |*shell*_*i*_–*shell*_*j*_|+|*sector*_*i*_–*sector*_*j*_|.

The parameter σ is an empirical value. According to experiments, the σ value can be set between 1 and 10. In this paper, we set the σ value at 5.0. The function d(i, j) represents the distance of the grids i and j. According to (2), the distance for two neighborhood histograms is determined not only by the difference of the grids in the same position but also by the adjacent grids whose effects are given in matrix A.

### C. Pattern Synthesis

With the histogram comparison algorithm described previously, we can search the sample pattern and find the matching element whose histogram is the most similar to that of the current extending element, i.e., the two histograms have minimum error. We add the neighboring elements of the matching element to the corresponding position in the synthesis area, and the synthesis pattern completes one extension. By repeating the steps above, we obtain our final synthesis pattern.

## Multiple Types of Element Synthesis

For the patterns in which elements have more than two types, a corresponding number of histograms must be used to record the neighborhood information for each type of element. We use the synthesis of two types as an example to illustrate our method.

**Fig 7:**
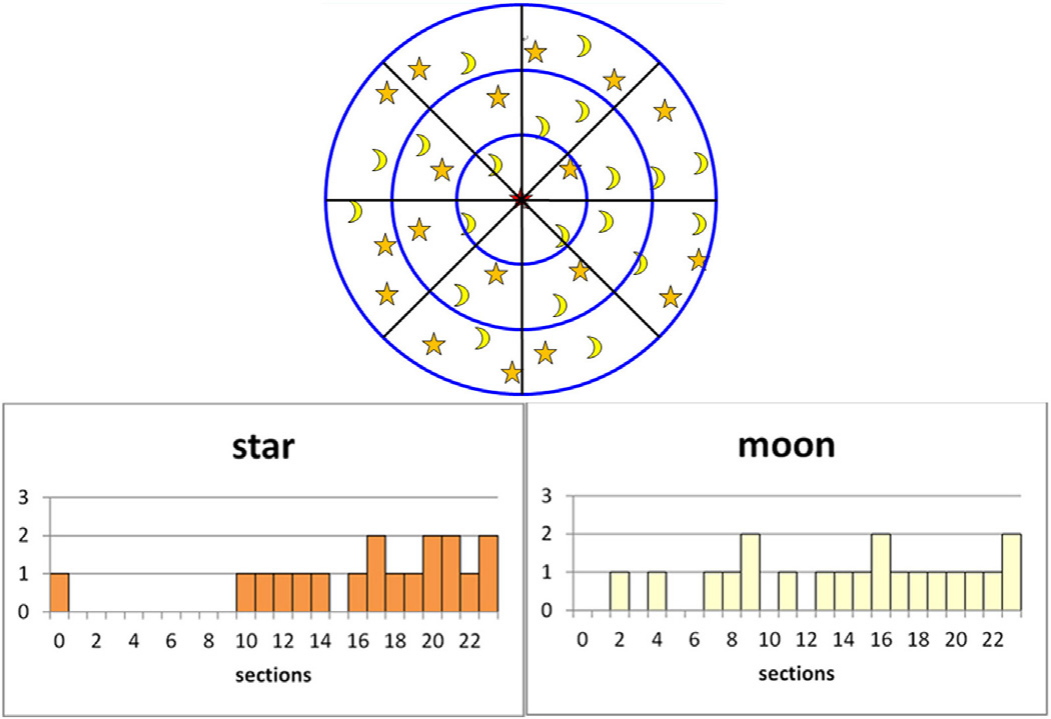
Multiple neighborhood histogram calculations.

We use two histograms to express the distribution of the two types of elements separately (shown in Fig 7). The histogram shown above represents the star-shaped element distribution and the one below represents the moon-shaped element distribution.

In the synthesis process, we apply a weighted sum formula to compute the neighborhood distance as follows:

$$d_{z}=w_{1}\cdot d_{1}+w_{2}\cdot d_{2}$$ (3)

*d*_*z*_ is the neighborhood distance; *d*_*1*_ and *d*_*2*_ are the distances of two types of histogram; *w*_*1*_ and *w*_*2*_ are the weights for *d*_*1*_ and *d*_*2*_. The element in the sample pattern that ensures the value *d*_*z*_ receives a minimum is the matching-element.

The weights are used to adjust the impact of each type of element; we note the phenomenon in which one type might play a leading role in the entire structure of the pattern, whereas others might only act as ornaments or background texture. In this case, we choose to amplify the weight of the main element type and decrease the weight of the unimportant types.

**Fig 8:**
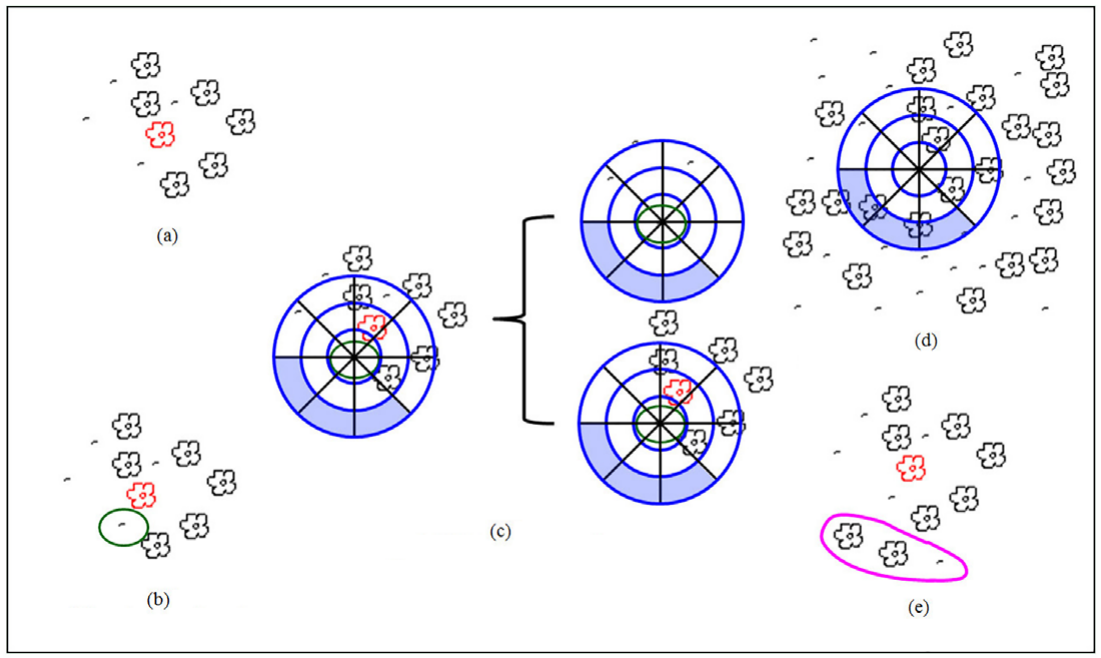
Vector pattern synthesis for multiple types of elements. (a) Initial synthesis pattern, in which the red element is the center element; (b) picking the extending element (the element in the green circle); (c) multi-histogram generation; (d) finding the matching element; (e) extending the synthesis pattern.

Similar to the single type of element, the neighborhood elements of the matching element are replenished in the synthesis pattern to accomplish one extension. The main processes are shown in Fig 8.

### Construction of Three-Dimensional Forest Scenes

The sample pattern represents the plants distribution of a small sample forest. The synthesis pattern represents the plants distribution of a larger area of forest. By giving the 2D plants distribution information, we can construct the 3D forest scenes by loading the 3D plant models.

**Fig 9:**
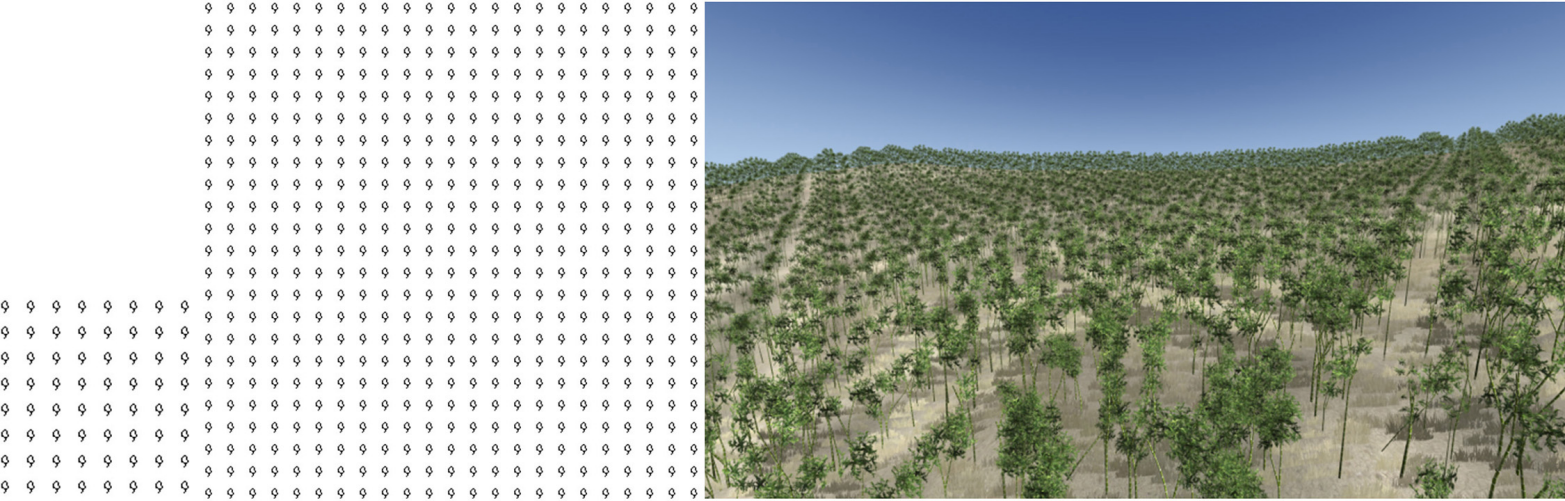
Regular distribution sample synthesis. Left: sample; Middle: synthesized result; Right: 3D forest scene.

**Fig 10:**
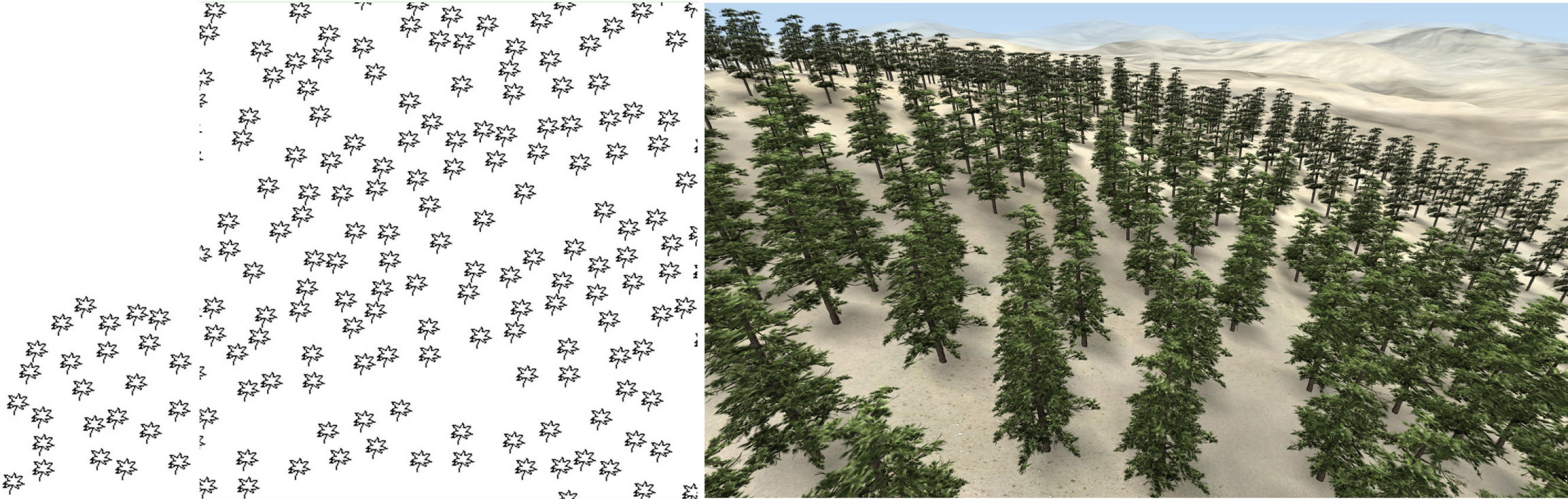
Random distribution sample synthesis. Left: sample; Middle: synthesized result; Right: 3D forest scene.

**Fig 11:**
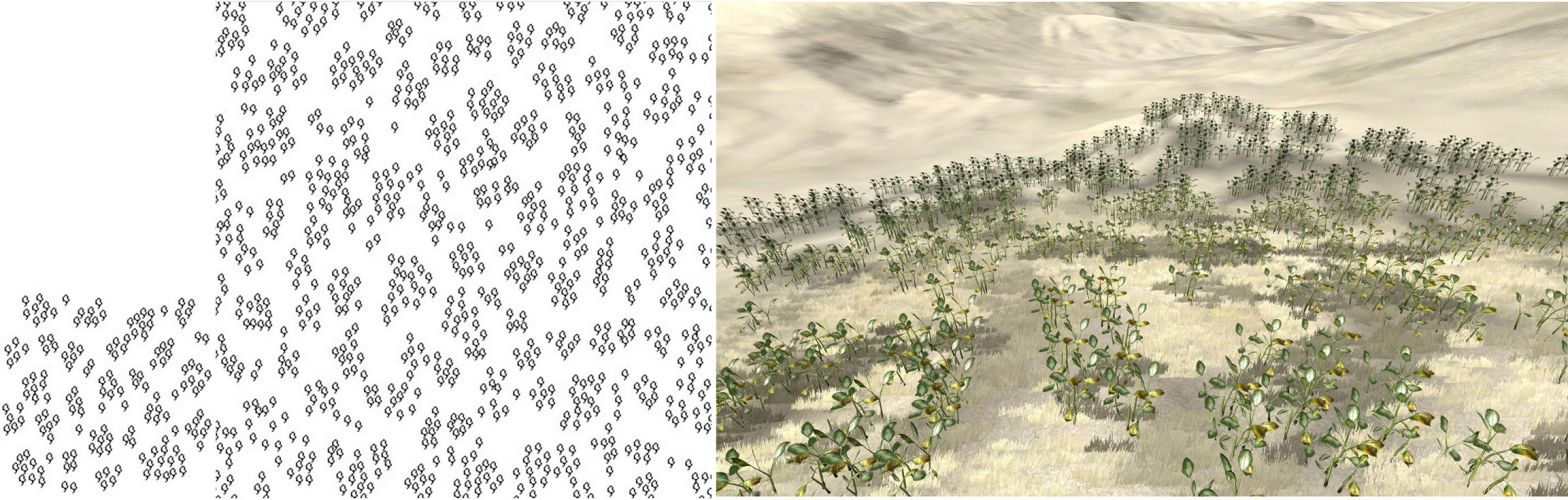
Clustered distribution sample synthesis. Left: sample; Middle: synthesized result; Right: 3D forest scene.

**Fig 12:**
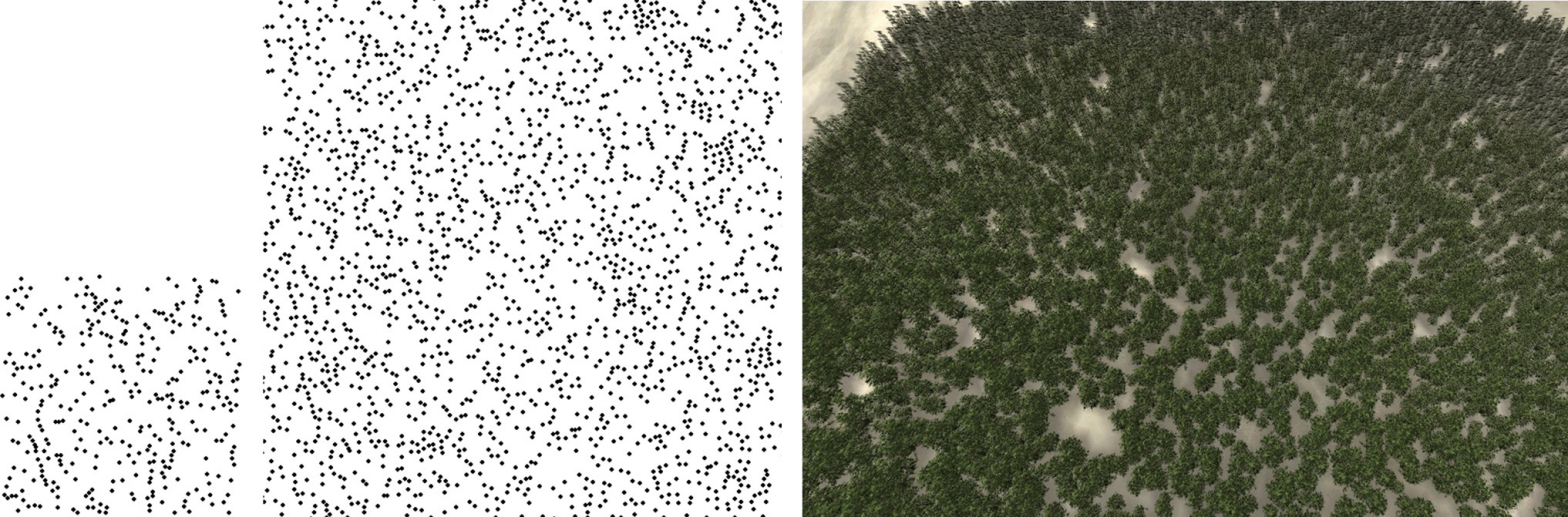
Synthesis of the sample obtained from a practical forest by field measurement. Left: sample; Middle: synthesized result; Right: 3D forest scene.

We build the 3D model for each type of plant, and position these plant models in the scene according to the distribution information in the 2D vector pattern. By using this manner, we constructed the 3D forest scenes (as shown in the right part of Figs 1 and 9–12).

In Figs 1 and 9–11, there are no DBH and height information, so we assign the size of each tree model by just disturbing the value randomly in a certain threshold. While, in Fig 12, the sample data contain the DBH, height and crown width of each tree obtained by field measurement. In synthesis process, each synthesized tree in the synthesis pattern can get its DBH, height and crown width from the corresponding one in the sample pattern. Therefore, in constructing the 3D forest scenes, these DBH, height and crown width data can be used to set the size of each tree model.

## Results and Discussion

### A. Results

Figs 1 and 9–12 show the synthesis results of five sample patterns and the 3D forest scenes constructed according to these synthesis patterns. The sample patterns in Figs 1 and 9–11 are designed interactively through our Pattern Design System. The sample data in Fig 12 is obtained by field measurement from a realistic Chinese pine forest at Fugou County, Henan Province, North China.

Fig 9 shows the regular distribution, Figs 10 and 12 show the distribution approximating to random distribution, and Fig 11 presents the clustered distribution. Our method is able to capture the characteristics in these sample distributions quite well and synthesize the appropriate results. Fig 1 includes two species of plants: arbor and shrub. An arbor is a commonly tall plant that displays a thick wooden stem and many large branches and always presents a sparse distribution because of competition for resources, whereas a shrub is a low several-stemmed woody plant that tends to distribute in aggregated patterns. Due to these features, the two species showed significant characteristics in the distribution, and the synthesized results reflect these characteristics quite well.

### B. Quantitative Evaluation

As discussed in section A, from the aspect of visual effects, the synthesis results appear to be similar to the sample pattern. However this is merely a sort of subjective judgment. We also try to seek some quantitative indices to evaluate the synthesis results in a more objective way.

In the field of forestry science, some indices are introduced to describe the trees’ distribution status, such as density and aggregation index. In this section, we will give a quantitative evaluation based on these two indices. By calculating the density and aggregation indices of sample data and those of synthesis data respectively, we succeed to tell if the synthesis results can preserve the distribution indices of the sample. The experimental data are shown in Tables 1 and 2.

**Table 1:** Density comparison.

	Sample(1×1)	Synthesis(3×3)	Synthesis(6×6)	Synthesis(10×10)	PCT. DEV.
Fig 9	64	64	64	64	0%
Fig 10	25	22.9	23.9	24.6	4.8%
Fig 11	165	172.2	165.8	166.2	1.86%
Fig 12	350	366.6	363	361	3.9%
Fig 1.**a**	172	169.5	168.3	172.2	1.2%
Fig 1.**b**	19	16.8	18.7	18.8	4.7%

**Table 2:** Aggregation index comparison.

	Sample(1×1)	Synthesis(3×3)	Synthesis(6×6)	Synthesis(10×10)	d	PCT. DEV.
Fig 9	-0.07885	-0.07891	-0.07885	-0.07887	0.29	0.03%
Fig 10	-0.008113	-0.008502	-0.008301	-0.008471	0.39	3.8%
Fig 11	0.06367	0.06083	0.06233	0.06425	0.19	1.9%
Fig 12	-0.002255	0.002277	0.002278	0.002280	0.1	1.0%
Fig 1	-0.001767	-0.001767	-0.001768	-0.001768	0.14	0.04%

In the experimental data, sample area is named as the unit area with an area of 1×1. We select three different areas of synthesis data for comparison with areas of 3×3, 6×6 and 10×10. For each sample, we firstly synthesize a very large area of distribution pattern (an area as large as 15×15), which is called M. After that, all the synthesis data will be selected from M for experimental comparison. For instance, to compute the indices of the synthesis pattern with an of area 3×3, we will randomly pick out 10 different patches with an area of 3×3 in M, then calculate the indices of these 10 patches, and finally figure out their average value as the index of the synthesis (3×3) in Tables 1 and 2.

The Ripley’s index [19] is adopted as the aggregation index in our experiments. In the Ripley’s index, if the index is less than 0, it means that the distribution tends to be a uniform one; if the index is larger than 0, the distribution tends to be an aggregation one; otherwise, the distribution is random. From Table 2, we can see that the sample in Fig 9 is a uniform distribution, the sample in Fig 11 is an aggregation distribution, and the samples in Figs 1, 10 and 12 are approaching the random distribution.

In Tables 1 and 2, PCT. DEV. is defined as the percentage of indices deviation between sample and synthesis data. If we suppose the indices of sample as *I*_*s*_ and synthesis result 3×3, 6×6 and 10×10 as *I*_*1*_, *I*_*2*_, *I*_*3*_, the PCT. DEV. D can be calculated as:

$$D=|((I_{1}+I_{2}+I_{3})/3-I_{s})|/I_{s}\times 100\%$$

There are 2 species in the sample of Fig 12. In Table 1, we compute the density for the two species respectively. While computing the aggregation index of Table 2, instead of distinguishing the species, we calculate the index for all the plants together.

From Tables 1 and 2, we can tell that the PCT. DEV. is less than 5% in all the examples, which is quite small. Hence from the perspective of the quantitative evaluation, our synthesis method is capable of preserving the distribution characteristics of samples in synthesis, at least practical in the indices of density and aggregation index.

### C. Comparison and Discussion

**Fig 13:**
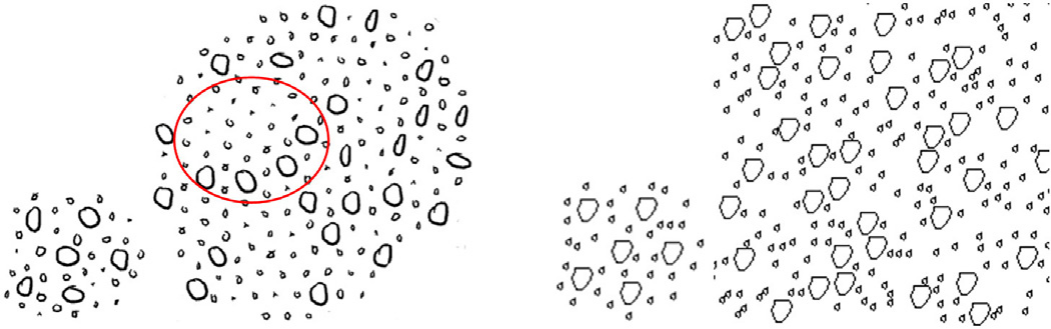
Comparison with Barla’s method. Left: Barla’s method; Right: Proposed method.

The explicit expression strategy used by Barla et al. [16] and Ijiri et al. [17] might cause error accumulation because the positions of corresponding neighborhood elements of the extending element and the matching element cannot exactly match in each extension, which is observed clearly from Fig 13(left). Our method uses an implicit method to record the distribution relationship by counting the number of neighborhood elements lying in a rough area (grids) and treating it as the neighborhood relationship. In this way, the neighborhood comparison has a type of fuzzy effect, which reduces the error accumulation in the synthesis process and might produce better results (shown in Fig 13 right).

Fig 13 shows a comparison of Barla’s method and the proposed method. It can be clearly observed that the synthesis pattern of Barla is in alignment and portrays the same space between elements, showing significant difference from the sample pattern (circled in red), whereas our result is more visually similar to the sample pattern.

Furthermore, the methods of both Barla et al. and Ijiri et al. must sort the neighborhood elements and calculate the Euclidean distance several times in each comparison. The optimization algorithm used by Ijiri et al. to improve the synthesis results further reduces the operation efficiency. In contrast, the histogram-based comparison method we propose must calculate the distance of the histograms only once, which reduces the calculation quantity and accelerates the operation. Table 3 shows that our method is quite fast even for synthesis of tens of thousands of plants, making it possible to apply this method in large-scale forest generation.

**Table 3:** Operational efficiency of the proposed method (second).

Type num	Sample elem num	Synthesis elem num	Analysis time cost	Overall time cost
**1**	50	500	0.015	0.76
**1**	70	1500	0.016	2.48
**2**	50	500	0.015	0.9
**2**	70	1500	0.016	2.73

## Conclusions and Future Work

We propose a vector pattern synthesis method for construction and simulation of a large-scale forest scene. Using the synthesis of the vector pattern, we propose a histogram-based strategy to express the topology neighborhood information. Our method can reduce the amount of multidimensional computation in the traditional neighborhood extraction method to one dimension; hence, this approach reduces the calculation quantity and accelerates the operation efficiency. Our method guarantees visual similarity between the synthesis pattern and the sample pattern and thus guarantees visual similarity between the generated virtual forest scenes and the real forest scenes.

In our synthesis method, we primarily consider the local distribution features of the sample pattern and do not take the overall statistical characteristics (i.e., density and probability distribution) into consideration. In future work, we will take the overall characteristics into account and generate vegetation distribution data that not only accurately reflect the local features but also conform to the overall features.
